# Characterizing metabolic responses of *Eleutherodactylus* frogs in Puerto Rico to different thermal treatments: implications for conservation

**DOI:** 10.1093/conphys/coag075

**Published:** 2026-10-06

**Authors:** Rafael Chaparro, Ana C Rivera-Burgos, Adam J Terando, Mitchell J Eaton, Jaime A Collazo, Eloy Martinez

**Affiliations:** North Carolina Cooperative Fish and Wildlife Research Unit, Department of Applied Ecology, North Carolina State University, 127 David Clark Labs, 100 Eugene Brooks Avenue, North Campus, Raleigh, NC 27695, USA; North Carolina Cooperative Fish and Wildlife Research Unit, Department of Applied Ecology, North Carolina State University, 127 David Clark Labs, 100 Eugene Brooks Avenue, North Campus, Raleigh, NC 27695, USA; U.S. Geological Survey, Southeast Climate Adaptation Science Center, North Carolina State University, 127 David Clark Labs, 100 Eugene Brooks Avenue, North Campus, Raleigh, NC 27695, USA; U.S. Geological Survey, Southeast Climate Adaptation Science Center, North Carolina State University, 127 David Clark Labs, 100 Eugene Brooks Avenue, North Campus, Raleigh, NC 27695, USA; U.S. Geological Survey, North Carolina Cooperative Fish and Wildlife Research Unit, Department of Applied Ecology, North Carolina State University, 127/226 David Clark Labs, 100 Eugene Brooks Avenue, North Campus, Raleigh, NC 27695, USA; Biological Sciences Department, Halmos College of Arts and Sciences, Nova Southeastern University, Parker Building, 3190 SW 74th Terrace, Fort Lauderdale/Davie Campus, Davie, FL 33314, USA

**Keywords:** Climate change, CO_2_ excretion, diel cycle*, Eleutherodactylus*energy budget, routine metabolic rate

## Abstract

Amphibians are increasingly vulnerable to extinction due to physiological stress induced by projected global warming and associated drying trends. Understanding the underlying mechanisms and consequences of these responses is crucial to developing effective conservation strategies. Puerto Rico hosts 15 endemic *Eleutherodactylus* frog species, which are particularly vulnerable to environmental changes due to poor vagility and narrow thermotolerance. In this study, we report routine metabolic rates (RMRs) of four species in response to four temperature treatments (15, 20, 25 and 30°C) at two temporal scales (24 and 72 hours) using indirect calorimetry. Results indicated that three species exhibited increased RMR with rising temperatures. The RMR at 25°C for *E. wightmanae* was spurious, and it was the only species that did not survive at 30°C. The Arrhenius breakpoint analysis indicated that *E. juanariveroi* RMR decreased between 25 and 30°C. In contrast, *E. antillensis* and *E. coqui* RMR increased with increasing assay temperatures. We discuss how diel metabolic demands decrease the scope at both thermal extremes for *E. antillensis* and *E. coqui*, and how habitat temperature may impinge energetic challenges to those coping with documented warming trends, such as *E. wightmanae*.

## Abbreviations

CT_max_, critical thermal maximum
RMR, routine metabolic rate
TPC, thermal performance curve
gWM, grams wet mass.

## Introduction

Ectotherms rely on environmental temperature to regulate physiological performance, making thermal sensitivity a central determinant of their ecology and resilience under climate change (Deutsch *et al.*, 2008; Hillman *et al.*, 2009; Sunday *et al.*, 2012). Across diverse taxa, metabolic rates often shift predictably with temperature, influencing energy budgets and activity patterns (Huey and Stevenson, 1979; Angilletta, 2009; Tattersall *et al.*, 2012). Despite these broad patterns, tropical ectotherms remain comparatively understudied, even though many already experience temperatures near their upper thermal limits and may be disproportionately affected by warming (Deutsch *et al.*, 2008; Scheffers *et al.*, 2012; Bestion *et al.*, 2015; Nowakowski *et al.*, 2017).

Amphibians are among the most vulnerable taxonomic groups to extinction worldwide. Listing of amphibians as threatened or endangered is far greater than any other vertebrate taxon (Stuart *et al.*, 2004; IUCN SSC Amphibian Specialist Group, 2024), with global warming and drying projections among the most important causes of these trends (Lertzman-Lepofsky *et al.*, 2020; Greenberg and Palen, 2021; Rebouças *et al.*, 2021; Luedtke *et al.*, 2023). Global warming is of particular concern in the tropics because almost one half of global amphibian diversity occurs in this region (Heinicke *et al.*, 2007; Hawley Matlaga *et al.*, 2021), and many tropical ectotherms already experience temperatures near their upper thermal limits (Deutsch *et al.*, 2008; Hillman *et al.*, 2009; Scheffers *et al.*, 2012; Sunday *et al.*, 2012; Bestion *et al.*, 2015; Nowakowski *et al.*, 2017).

Current approaches to integrate both ecological effectors and studies of physiological limits of ectotherms are critical components to assess the “winners and losers” of global warming (Somero, 2010). However, while most studies on anuran metabolism have focused on temperate species, tropical anurans and ectotherms, in general, have been underrepresented in the scientific literature (Alves-Ferreira *et al.*, 2022). This underrepresentation is alarming given that the tropics harbour exceptionally high amphibian diversity and endemism, including the Caribbean archipelago, which contains >80% of species in the genus *Eleutherodactylus* (Dugo-Cota *et al.*, 2019; Hedges *et al.*, 2019). Puerto Rico harbours 18 *Eleutherodactylus* species of which 15 are endemic (Ríos López and Heatwole, 2023; Ríos-López *et al.*, 2025). Of these, three are listed as endangered (*E. jasperi*, *E. juanariveroi*, *E. cooki*), and 12 are considered at risk of becoming endangered according to the IUCN Red List of Threatened Species. The conservation of *Eleutherodactylus* frogs is of interest because they are valued culturally and play a role in ecosystem function through complex trophic interactions (Dodd and Seigel, 1991; Joglar, 1998; Beard *et al.*, 2002; Beard, 2007; Rohman *et al.*, 2020).

The vulnerability of *Eleutherodactylus* frogs in Puerto Rico, and throughout their range in the Neotropics, is rooted in their low vagility, high philopatry, and sensitivity to extremes in temperature and humidity (Luedtke *et al.*, 2023; Ríos López and Heatwole, 2023). These traits gain attention because available climatological models forecast a warming, drying trend for Puerto Rico and the region (Neelin *et al.*, 2006; Pulwarty *et al.*, 2010; Karmalkar *et al.*, 2013; Khalyani *et al.*, 2016; Bowden *et al.*, 2021). Previous work on the effects of temperature on *Eleutherodactylus* frogs in Puerto Rico has focused on thermal thresholds (e.g. critical thermal maximum [CT_max_]; Christian *et al.*, 1988; Chaparro *et al.*, in review; Delgado-Suazo and Burrowes, 2022), as these define the ambient conditions where frogs will fail to meet self-maintenance functions (Andrade, 2016). However, less apparent, but equally important are the sub-lethal effects of changing environmental conditions on metabolic rates and energy budgets (Blaustein *et al.*, 2010). For example, recent work with *E. coqui* at El Yunque National Forest showed that coquis increased their locomotor activity in response to a 4°C increase in ambient temperature as some individuals searched for thermal refugia (Hawley Matlaga *et al.*, 2021).

Thermal performance curves (TPCs) provide valuable insights into how ectotherms’ metabolic demands shift with body temperature (Huey and Stevenson, 1979; Angilletta, 2009; Tattersall *et al.*, 2012; Andrade, 2016, 2021; Seebacher and Little, 2021; Kefford *et al.*, 2022). TPCs are often derived using indirect calorimetry to measure the routine metabolic rate (RMR), which represents the energy expenditure required for maintaining vital functions, while accounting for spontaneous locomotor activity in a post-absorptive state (Hillman *et al.*, 2009). These measurements are critical for predicting an organism’s caloric requirements across varying thermal environments, making TPCs invaluable tools for assessing ecological vulnerability (Ríos-Franceschi *et al.*, 2019).

Here we characterized the effects of temperature on both nocturnal and diurnal RMR (henceforth diel RMR) for *E. antillensis* and *E. coqui*, and characterized the performance curve (TPC) of RMR for *E. antillensis*, *E. coqui*, *E. juanariveroi,* and *E. wightmanae*. We hypothesized that diel RMR of *E. antillensis* and *E. coqui* would be comparably higher during the night due to the species’ nocturnal feeding habits, reflecting circadian rhythms, as metabolic rates are known to vary in response to environmental factors such as photoperiod and temperature. By evaluating diel RMR across various temperature treatments, we sought to identify the differences in diurnal versus nocturnal activity, and the role of temperature affecting the scope between these periods.

We also hypothesized that the TPCs for the mostly highland-distributed *E. coqui* would be more sensitive to thermal regimes compared to the mostly lowland-distributed *E. antillensis*. This is because high-elevation species typically operate within a narrower range of environmental conditions compared to widely distributed species (Joglar *et al.*, 1996; Joglar, 1998; Rivera-Burgos, 2019; Ríos López and Heatwole, 2023). Additionally, we hypothesized that *E. juanariveroi*, which inhabits exclusive lowland freshwater wetlands, would exhibit TPCs comparable to other generalists whose range include lowlands (e.g. *E. antillensis*). We also assessed whether *E. antillensis*, *E. coqui,* and *E. juanariveroi* exhibited a change of RMR as a function of treatment temperatures (e.g. 15, 20, 25, and 30°C). We addressed this question in two ways: (i) estimating the Q_10_ for three temperature intervals, and (ii) estimating the Arrhenius breakpoint in temperature (ABT). We used two metrics to guard against spurious inferences using only four least squares mean (LSM) mass-specific VCO_2_ estimates. The Q_10_ is a simpler but informative approach to assess how metabolism changes as temperature increases (Schulte, 2015; Mundim *et al.*, 2020). Lastly, we plotted RMRs for *E. antillensis* and *E. coqui* along the range of treatment temperatures in this work and then overlaid values of occupancy probability estimated along altitudinal gradients in the west-central mountains of Puerto Rico (Rivera-Burgos *et al.*, 2021)*.* Our motivation with the data integration is to explore how the metabolic demands of two *Eleutherodactylus* species align to their distribution, exemplifying how data integration could inform research to foster conservation efforts.

## Materials and methods

### Focal species

This study focused on *Eleutherodactylus antillensis*, *E. coqui*, *E. juanariveroi*, and *E. wightmanae*, chosen for their varied elevation ranges, habitat associations, and body mass. These species represent a range of ecomorphs reported for the genus (Dugo-Cota *et al.*, 2019), distinguished by their morphology (e.g. body mass) and habitat occurrence. These adaptive traits are prominently used to gain insights about temperature-driven physiological rates as compared to phylogenetic relationships (Luedtke *et al.*, 2023; Nori *et al.*, 2023). *Eleutherodactylus wightmanae* is a leaf-litter ecomorph, representative of eight other high-elevation species (*E. portoricensis, E. richmondi, E. gryllus, E. hedricki, E. enaidae, E. jasperi, E. locustus, and E. unicolor*). *Eleutherodactylus coqui*, an arboreal and bromelicolous ecomorph, is widespread but primarily found in higher, cooler environments (Beuchat *et al.*, 1984; Rivera-Burgos *et al.*, 2021). In contrast, *E. antillensis*, a terrestrial ecomorph, is typically found in lower elevation areas with higher temperatures and lower humidity (Rivera-Burgos *et al.*, 2021). Lastly, *E. juanariveroi*, an endangered species, is associated with lowland freshwater swamps (Ríos-López *et al.*, 2014).

### Specimen collection

Four species of *Eleutherodactylus* frogs were surveyed across west-central Puerto Rico between 2021 and 2023, each collected from distinct ecological settings. *E. antillensis* was sampled at Hacienda Ana Luisa in Las Marías (18.2103, −67.00087; 526 m), representing a mid-elevation site in the island’s interior. *E. wightmanae* was collected at Parque Ecológico in Monte del Estado, Maricao (18.14353, −66.97875; 879 m), the highest-elevation locality in the dataset. *E. juanariveroi* was obtained from a wetland in Caño Tiburones, Arecibo (18.46105, −66.67182; 3 m), a low-lying coastal habitat (Fig. 1). Relative humidity and ambient temperature were measured at all collection sites using a pocket weather meter (Kestrel 3500 Pocket Weather Meter, Nielsen-Kellerman, Boothwyn, PA, USA; accuracy ±1°C, ±3%, respectively). Individuals were collected by hand, placed in 473 ml food-grade polypropylene cups with sterile napkins and dechlorinated water, and transported to the NCSU field lab in Las Marias (18.21049, −67.00166; Fig. 1) following institutional animal care and use committee (IACUC) approved protocol # 19-028-O, Department of Natural and Environmental Resources permit # 2022-EPE-003, and USFWS threathened/endangered (TE) permit TE 697819-5. Specimens were held in 15 L containers at a mean laboratory temperature of 22.13 ± 0.96°C (± standard deviation; SD) under natural diel light–dark cycles for a minimum of 48 hours to reduce handling stress and to monitor signs of illness or abnormal behaviour. This period also allowed gut voidance and ensured a post-absorptive state before trials. Each container was lined with sterile napkins, misted with dechlorinated water, with daily cleanup to reduce chances of waste accumulation.

**Figure 1:**
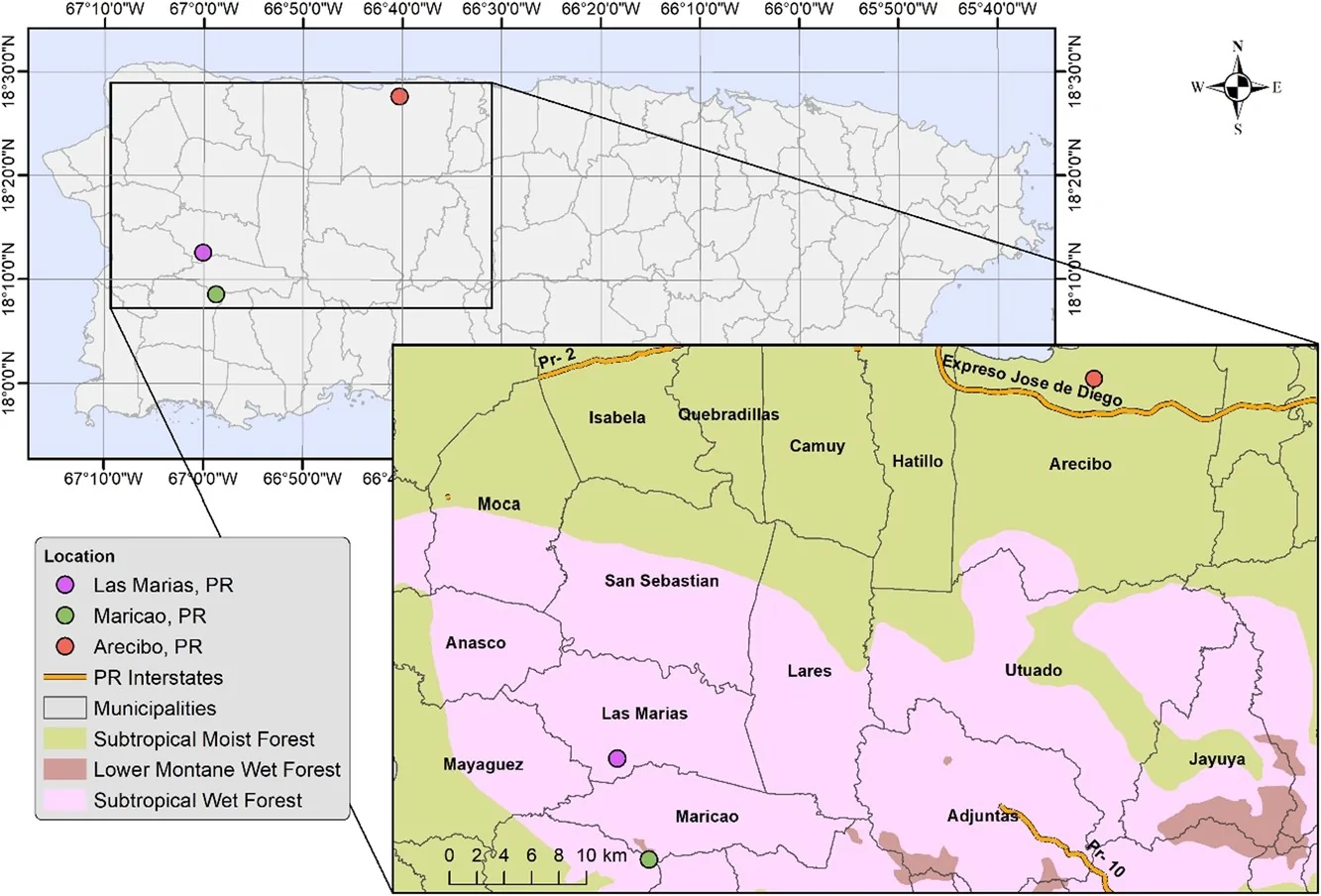
Map of Puerto Rico depicting the locations (inset) where samples of four species of *Eleutherodactylus* were collected. *E. juanariveroi* was collected in north-central wetlands, and the remaining species (*E. coqui*, *E. wightmanae*, and *E. antillensis*) were collected ≥500 m in the municipalities of Maricao and Las Marías

Temperatures in Puerto Rico have increased almost 2°C since 1950 (Runkle *et al.*, 2022), with increases occurring mostly during nighttime with temperatures above 26.6°C since 2000. Puerto Rico is projected to have an increase in average temperature of 1.5°C (range 1.2–1.7°C) and drier conditions by 2060 (Bowden *et al.*, 2021). Las Marias and neighbouring towns (e.g. Maricao) are characterized by daily highs that rarely exceed 30°C or fall below 16°C (https://weatherspark.com/y/27782/Average-Weather-in-Maricao-Puerto-Rico). Humidity is consistently high, exceeding 70%. Total precipitation ranges from 1950 to 2160 mm per year, with the wettest month being September. In contrast, Arecibo is exposed to temperatures ranging from 21 to 32°C throughout the year. Data since 1970 indicate that average annual highs are around 31.6°C and lows of 22°C. Average annual precipitation is 88.9 mm, with higher amounts in May and lower in winter (February–March). Average humidity is 77% (https://rcc.cimh.edu.bb/).

### Thermal performance curve of routine metabolic rates

Twenty-eight individuals from each focal *Eleutherodactylus* species were randomly split into four groups of seven. Each group was exposed to one of four temperature treatments (15, 20, 25, and 30°C). Temperature control of both instrumentation and specimens was carried out by means of a walk-in temperature control chamber as described by Martinez and Agosta (2016) with minor modifications. Average within-trial stability (SD) for 15°C was 0.09, 0.06 for 20°C, 0.02 for 25°C, and 0.02 for 30°C (*n* ranged from 233 to 350 trial runs). The range of treatment temperatures includes the minimum and maximum long-term temperatures recorded in Maricao/Las Marias and Arecibo. After the 48-hour holding period described above, all specimens were weighed to the nearest milligramme using an analytical balance (VWR-123P, VWR International, Radnor, PA, USA), then placed immediately into individual 30 ml modified syringes used as respirometry chambers. While inside the chamber, data collection started one hour after placing specimens in the chamber, to reduce the handling effect on the individuals. Experiments were initiated between 18 and 21 hours, conducted during the warm-wet season (May–November; Delgado-Suazo and Burrowes, 2022). Once field laboratory conditions were set at a particular temperature treatment, we ran experiments for all species, then repeated the process for the subsequent treatments. We monitored metabolic gases under saturated water vapour conditions for 24 hours for each treatment.

### Diel variation in RMR and experimental temperature

We conducted trials to assess diel patterns in RMR (diel RMR) for thermotolerant species *E. coqui* (CT_max_: 38.10 ± 0.24) and *E. antillensis* (CT_max_: 39.55 ± 0.49; Chaparro *et al.*, in review). During these trials, RMR was recorded at 3-hour intervals while specimens were maintained under their normal diel light–dark cycle schedule without any manipulation. Specimens were held at controlled environmental conditions, with RMR data collected using respirometry chambers housed in the temperature-controlled setup described in the previous section. All experiments were run with a standardized minimum relative humidity of 95%.

### Analysis of water vapour and respiratory gases

Each experimental group was exposed to four temperature treatments, with each run including an empty chamber for signal baseline determination and seven specimens per treatment, housed individually in 30 ml sealed chambers (Fig. 2). The chambers were connected to an eight-channel multiplexer (Sable Systems International, Las Vegas, NV, USA; MF-8) controlled by the Field Metabolic System (FMS) using a universal interface (Sable Systems; UI). Sampling was programmed via Sable Systems’ ExpeData software to select chambers at timed intervals.

**Figure 2:**
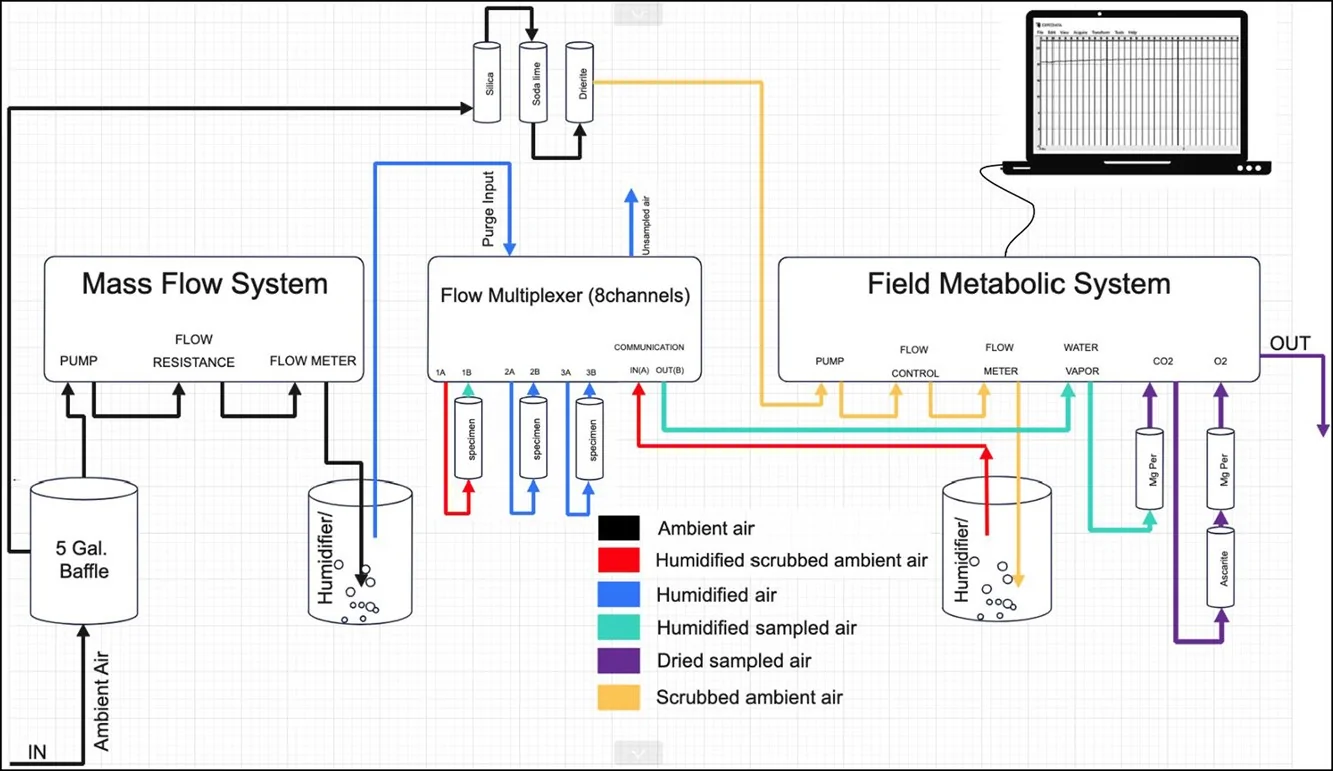
Diagram of a Sable Systems FMS, configured in flow-through, push mode, used to measure rates of CO_2_ production in four species of *Eleutherodactylus* frogs in this study

The MF-8 routed air from the selected chamber to the FMS for gas analysis. A centrifugal pump powered the air circuit, delivering CO_2_- and H_2_O-free air to the system, which was re-humidified by a customized dew point generator to 2–3°C below assay temperature. This air was then routed to the selected chamber, and respiratory gases were analysed.

Gas analysis involved sequential measurements of water vapour pressure (WVP) and CO_2_. Excurrent air was first routed to the WVP sensor, then dried with magnesium perchlorate before CO_2_ analysis. After CO_2_ scrubbing with Ascarite and magnesium perchlorate, O_2_ fractional composition was measured in the dry, CO_2_-free air. Inert tubing (1/8" ID, Cole-Palmer Bev-a-Line) routed air between chambers and sensors. Specimens were sampled for 10 minutes at 1.5-hour intervals during diel RMR trials and 15 minutes at 2.25-hour intervals during TPC RMR trials. Baseline chambers were sampled twice per interval to monitor signal drift. Data collection was automated over 24-hour periods for TPC trials and 72 hours for diel RMR trials.

Unselected chambers received a continuous 30 ml/min airflow from a mass flow generator (MFS; Sable Systems), keeping conditions stable without CO_2_ enrichment or O_2_ depletion. The setup was housed in a temperature-controlled environment maintained by an independent air conditioning unit (Martinez and Agosta, 2016), ensuring stable and programmable temperature regimes for calibration and trials.

### Data processing

The Diel RMR and TPC experiments utilized an ExpeData Macro Utility to convert sampled gases from percent composition to molar concentration, as previously described (Lighton, 2018). This was accomplished using the following equations:

Water vapour VH_2_O = FR × (FeH_2_O − FiH_2_O)/(1 − FeH_2_O)

To calculate the rate of water vapour exchange, where VH_2_O represents the rate of water vapour exchange, FR is the flow rate of gas, FeH_2_O is the fraction of water vapour in the exhaled gas (Fe is fractional excurrent), and FiH_2_O is the fraction of water vapour in the inhaled gas (Fi is fractional incurrent).

Carbon dioxide VCO_2_ = FR × (FeCO_2_ − FiCO_2_)/(1 − FeCO_2_)

To calculate the rate of carbon dioxide exchange, where VCO_2_ represents the rate of carbon dioxide exchange, FR is the flow rate of gas, FeCO_2_ is the fraction of carbon dioxide in the exhaled gas, and FiCO_2_ is the fraction of carbon dioxide in the inhaled gas.

The equations account for fluctuations in the FR and the fractional composition of gases (incurrent or excurrent, in relation to the chamber) into the baseline chamber and each specimen chamber. The FiH_2_O and FiCO_2_ values were obtained from the baseline chamber, while the FeH_2_O and FeCO_2_ values correspond to the flow of gases from the specimen’s chamber. As the specimens required incoming air to be saturated with water vapour, WVP measurements were not further processed for analysis. CO_2_ values were used as the proxy for metabolic activity, as the sensor type (laser beam) is significantly more thermally stable than the O_2_ sensor (paramagnetic O_2_ cell) in the FMS. See supplementary materials for macro scripts used in this study.

### Data analysis

We used a split-plot mixed analysis of variance (ANOVA; JMP Pro 17, version 13.2.1; SAS Institute, Cary, NC), a univariate analytical framework suitable for repeated measures data, to analyse data from TPC RMR and Diel RMR experiments. For TPC RMR analyses, data for *E. antillensis*, *E. coqui*, and *E. juanariveroi* were analysed together to determine if there were interspecific differences in responses. The response variable was mass-specific VCO_2_ (μl/min). We used a mass-adjusted response variable due to a positive relationship between excretion of VCO_2_ and body mass (repeated measures analysis of covariance; ANCOVA, *P* < 0.05). Model terms were species (plot), assay temperature (hereafter treatment) as the sub-plot, and individuals within species with treatment chamber treated as a random effect. The model also included a “time” term that accounted for the fact that mass-specific VCO_2_ data represented a repeated measure (i.e. measurements taken at 2.25-hour intervals after starting the experiment) and relevant interactions (e.g. species × assay temperature, species × time, species × assay temperature × time). Data were log-transformed to meet the homogeneity of variance assumption (O’Brien *P* > 0.05). *E. wightmanae* data were analysed separately with a mixed model. The fixed effect was treatment (assay temperature), and we used an unstructured variance, with repeated measures taken over time and the frog specimen being the subjects. The reason for this was that: (i) excretion rates at 25°C were not reliable based on thermodynamic effects of physiological processes, and (ii) none of the experimental specimens tolerated 30°C. As with the other species, the remaining data (15 and 20°C) were log-transformed to meet the homogeneity of variance assumption (Chi-square = 6.37, df = 5, *P* = 0.27).

For Diel RMR analyses, data for *E. antillensis* and *E. coqui* were combined to assess species differences, using repeated measure values every 3 hours for 72 hours. In this model, we included the diel term (day/night) to examine diurnal vs. nocturnal cycles on the metabolic rates of focal species at various temperature treatments. If model terms of interest were significant, we ran customized *F*-tests (orthogonal contrasts) between means of interest to determine if they differed significantly. Data were log-transformed (ln mass-specific VCO_2_) to meet the homogeneity of variances by species (O’Brien test, *P* > 0.05).

We scaled mass-specific VCO_2_ (ln rate × 100) for ABT analyses, and converted the independent variable as the reciprocal of the absolute temperature (1000/T). We plotted results after selecting the best model to describe the relationship between excretion rate and treatment temperatures by comparing support Akaike Information Criterion (AIC) between a linear or non-linear (piecewise) regression. We estimated Q_10_ by estimating LSM excretion rates for each of the experimental temperatures (i.e. one-way ANOVA). We used these values to estimate Q_10_ as (r2/r1)^(10/(t2 − t1)), where r1 and r2 are pairs of LSMs, and t1 and t2 correspond to one of the three pairs of intervals in the dataset. Estimates of standard error were obtained using the Delta Method. All analyses were done with JMP Pro 17 (version 13.2.1; SAS Institute, Cary, NC).

We summarized all results using LSM mass-specific VCO_2_ instead of raw means because statistical inference using LSM is more reliable when models are complex and data are unbalanced. For example, sometimes our respirometer did not record reliable data at every time interval (e.g. transcription error). Also, in some instances, we got data from only 6 experimental tubes rather than 7 for both TPC and Diel RMR experiments due to having to omit a tube where some frogs excreted or equipment malfunction. We report goodness-of-fit (e.g. homogeneity tests) for tests, and whether values were log-transformed (ln rate).

### Data integration

We plotted ln LSM mass-specific VCO_2_ for *E. antillensis* and *E. coqui* along the range of treatment temperatures in this work and then overlaid values of occupancy probability estimated along altitudinal gradients in the west-central mountains of Puerto Rico (Rivera-Burgos *et al.*, 2021)*.* In contrast to the aims of Rivera-Burgos *et al.* (2021), we focused on providing a distributional context to physiological results. Estimates of occupancy were obtained using presence/non-presence data (i.e. 1 or 0, respectively) obtained using acoustic recorders (Rivera-Burgos *et al.*, 2021). Data collection also included physical (e.g. temperature) and habitat covariates (e.g. vertical and horizontal cover). Data were analysed using a multi-season, multi-state occupancy model (MacKenzie *et al.*, 2006). Occupancy is defined as the probability of detecting a species of interest at a sampling location (e.g. survey station), given that the species is available to be detected (MacKenzie *et al.*, 2006).

## Results

### Body mass and environmental conditions at capture

The average body mass for *E. wightmanae* was 0.67 ± 0.005 g and 0.29 ± 0.003 g for *E. juanariveroi*, both used for TPC RMR experiments. The body mass of *E. coqui* and *E. antillensis* used in Diel RMR experiments was 2.15 ± 0.040 g and 1.32 ± 0.020 g, respectively. Body mass of frogs used in TPC RMR experiments was 2.16 ± 0.050 g (*E. coqui*) and 1.38 ± 0.020 g (*E. antillensis*). Air temperatures were recorded every time frogs were collected for experiments at field locations. For *E. antillensis*, air temperature (°C) and relative humidity (%) averaged 23.57°C ± 0.49 and 84.47% ± 1.55; for *E. coqui* it was 23.18°C ± 0.61 and 83.48% ± 2.14; for *E. wightmanae* it was 22.04°C ± 0.72 and 90.55% ± 2.19; and for *E. juanariveroi* it was 26.55°C ± 0.25 and 92.66% ± 0.21, respectively.

### Diel RMR

In this study, we found that RMR did not differ significantly among species, diel periods, or temperature treatments (*F* = 2.35; df = 3, 1199; *P* = 0.07; *R*^2^ = 0.69). However, the analysis highlighted two important patterns of biological significance (Fig. 3). As hypothesized, RMR increased with temperature treatments (*F* = 41.06; df = 3, 46; *P* < 0.001). In both species, the highest metabolic demands corresponded with evening hours (F = 34 317.65; df = 3, 1199; *P* < 0.001).

**Figure 3:**
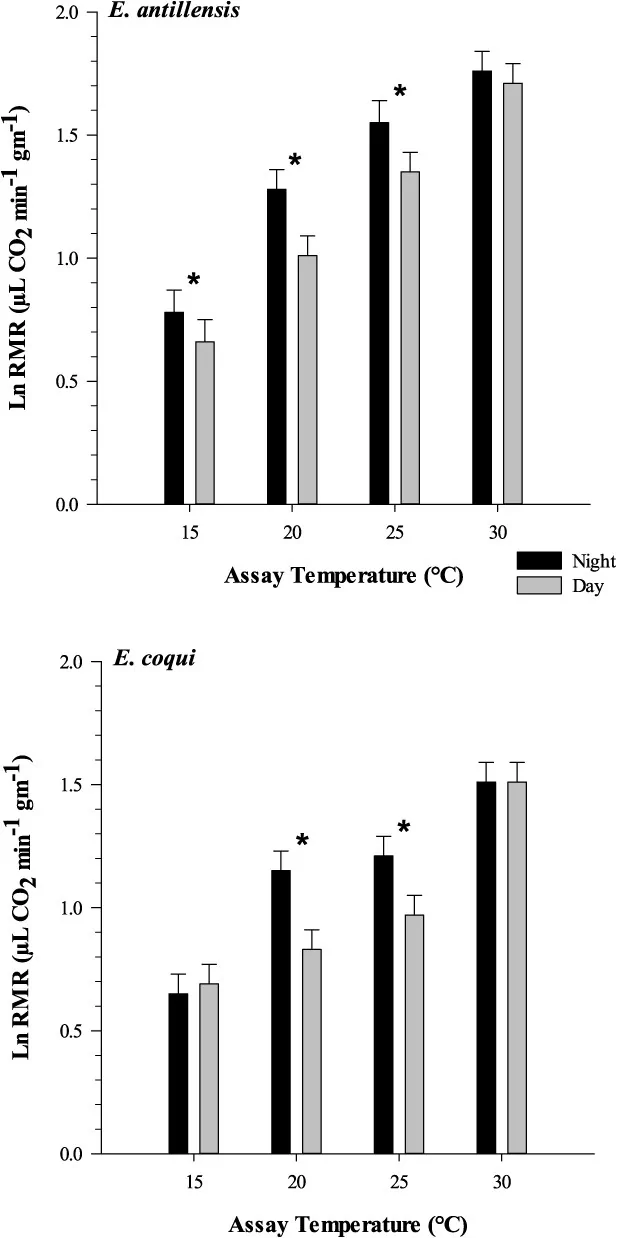
Diel changes in routine metabolism (μl CO_2_/min grams wet mass [gWM]); RMR) of *E. antillensis* and *E. coqui* collected from west-central Puerto Rico. RMR was measured for 72 consecutive hours at the temperatures shown. Asterisks (*) indicate significant differences between the diurnal and nocturnal RMR values at the corresponding assay temperature. Each data point is shown as the log-transformed least-squares mean RMR ± standard error (SE; *n* = 7)

### TPC RMR

Metabolism during TPC RMR experiments differed among *E. antillensis*, *E. coqui*, and *E. juanariveroi* by time and temperature treatment (*F* = 4.04; df = 54, 637; *P* < 0.001; *R*^2^ = 0.74; Fig. 4). Metabolic rates generally increased as experimental temperatures increased to 25°C but were markedly variable among species at 25°C (Fig. 4). In the case of *E. coqui*, routine metabolic demands increased with temperature, although the rates found at 20–25°C were statistically similar (*F* = 0.11, df = 1, 70; *P* = 0.73). In the case of *E. juanariveroi*, metabolism increased with assay temperature up to 25°C, but decreased at 30°C (*F* = 5.04, df = 1, 70; *P* = 0.03; Fig. 4). Conversely, *E. antillensis* RMR increased from 15 to 20°C, then decreased at 25°C, followed by a further increase at 30°C (*F* = 5.62, df = 1, 70; *P* = 0.02). Metabolic rates of the high-elevation, cold-adapted *E. wightmanae* varied by assay temperature as hypothesized (*F* = 21.60; df = 1, 47; *P* < 0.001). Interestingly, rates were higher at 15°C (1.52 ± 0.09) than at 20°C (0.88 ± 0.10). At 25°C, metabolic rates were not reliably measured, and at 30°C, none of the specimens survived the treatment.

**Figure 4:**
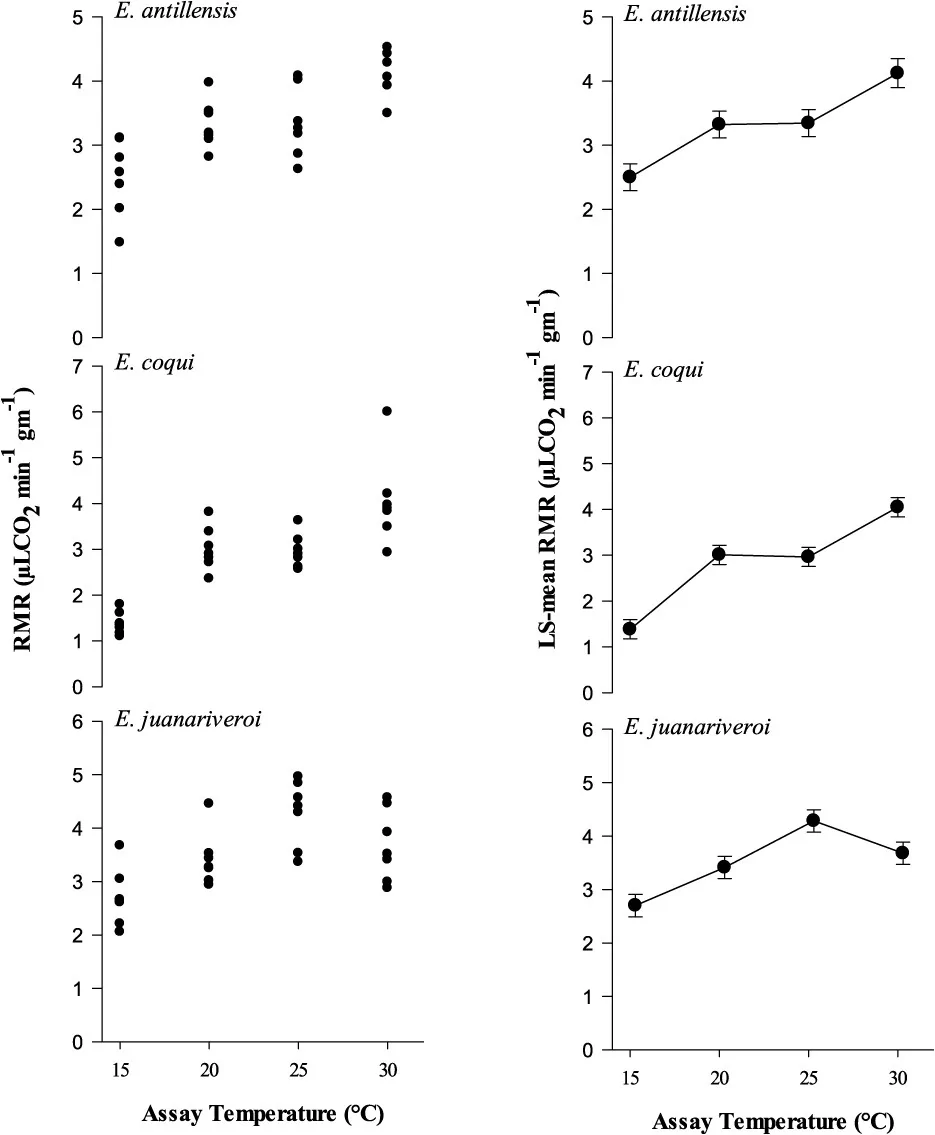
RMR measurements of individual specimens (*n* = 7) as a function of assay temperature for *E. antillensis*, *E. coqui*, and *E. juanariveroi* collected from west-central Puerto Rico (left panels). Right panels represent the least squares mean ± SE for each temperature treatment. Data points within each assay temperature were offset to improve the visibility of error bars

### Arrhenius breakpoint in temperature and Q_10_

There was weak support for a non-linear model (i.e. piecewise regression) to describe the relationship between RMR and treatment temperatures for *E. antillensis* (delta AIC = 0.39) and *E. coqui* (delta AIC = 3.09). Conversely, the support for a non-linear model was strong for *E. juanariveroi* (delta AIC = 34.66). Data for all models, linear or piecewise, met the homogeneity of variance assumption (*E. antillensis* Shapiro–Wilk = 0.83, *P* = 0.17; *E. coqui* S–W = 0.84, *P* = 0.20; *E. juanariveroi* S–W = 0.83, *P* = 0.17). Metabolic rates of *E. antillensis* (*y* = 5.22 + 0.03 × °C; *R*^2^ = 0.59) and *E. coqui* (*y* = 4.12 + 0.06 × °C; *R*^2^ = 0.82) increased linearly throughout the range of treatment temperatures (Fig. 5). In contrast, the ABT for *E. juanariveroi* was estimated to be 25°C (Intercept = 4.83, first slope = 0.05, change in slope = −0.10, break point = 24.95°C; *R*^2^ = 0.99; Fig. 5), indicating a reduction in metabolism with increasing assay temperatures.

**Figure 5:**
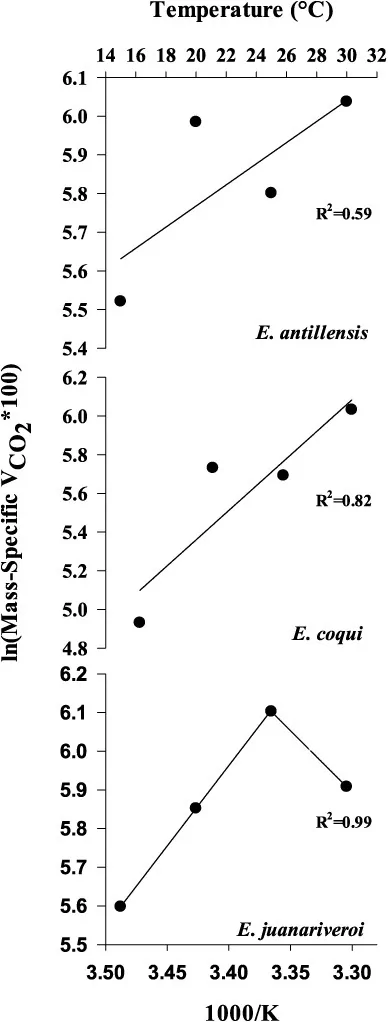
Log-transformed routine metabolism (μl CO_2_/min gWM) in *E. antillensis*, *E. coqui*, and *E. juanariveroi* from west-central Puerto Rico (black line). Lines represent either regression lines (*E. antillensis*, *E. coqui*) or an Arrhenius breakpoint calculated through a piecewise regression (*E. juanariveroi*), along with their coefficients of determination. The bottom x-axis represents the reciprocal of absolute temperature in kelvins typically used to represent the Arrhenius breakpoint, and the top *x*-axis represents the temperature in degrees Celsius for reference purposes

Changes in the RMR with temperature were assessed through Q_10_ calculations, which varied with temperature (Table 1). The highest and lowest Q_10_ values were found between 15–20°C and 20–25°C for *E. coqui* and *E. antillensis*, respectively. The highest Q_10_ values, indicating a stronger RMR response to temperature, occurred between 15–20°C and 25–30°C for *E. juanariveroi*. Values decreased during the last interval (25–30°C; Table 1).

**Table 1:** Reaction norm of RMR with temperature, calculated as Q_10_, for *Eleutherodactylus coqui*, *E. antillensis*, and *E. juanariveroi* in Puerto Rico

Species	Interval	Q_10_ (±SE)
*E. coqui*	15–20	4.96 ± 1.10
	20–25	0.92 ± 0.12
	25–30	1.97 ± 0.23
*E. antillensis*	15–20	2.52 ± 0.44
	20–25	0.71 ± 0.11
	25–30	1.57 ± 0.24
*E. juanariveroi*	15–20	1.66 ± 0.22
	20–25	1.65 ± 0.17
	25–30	0.67 ± 0.07

Q_10_ (±SE) values are reported for three temperature intervals defined by experimental treatments (15, 20, 25 and 30°C).

### Integrating TPC RMR and occupancy

The resulting integration of RMR as a function of temperature and occupancy data is summarized in Fig. 6. The integration revealed that *E. antillensis* and *E. coqui* could increase routine metabolic demands with increasing temperature, and to some degree, irrespective of their altitudinal distribution. Species are contrasting because *E. antillensis* occurrence is higher at low elevations compared to *E. coqui,* which has increased occurrence at high elevations. Occupancy probabilities for *E. antillensis* ranged from 0.75 to 0.90 at sea level–300 m, whereas occupancy probabilities for *E. coqui* ranged from 0.90 to 1.00 at 600–1100 m. Interestingly, the largest number of occupancy probability values align to those intermediate temperature regimes in our study. These temperature ranges also correspond to the lowest Q_10_ values found for RMR in this study. Additionally, occupancy temperatures align with the temperatures where both species displayed a scope of RMR between diurnal and nocturnal activities (Fig. 3).

**Figure 6:**
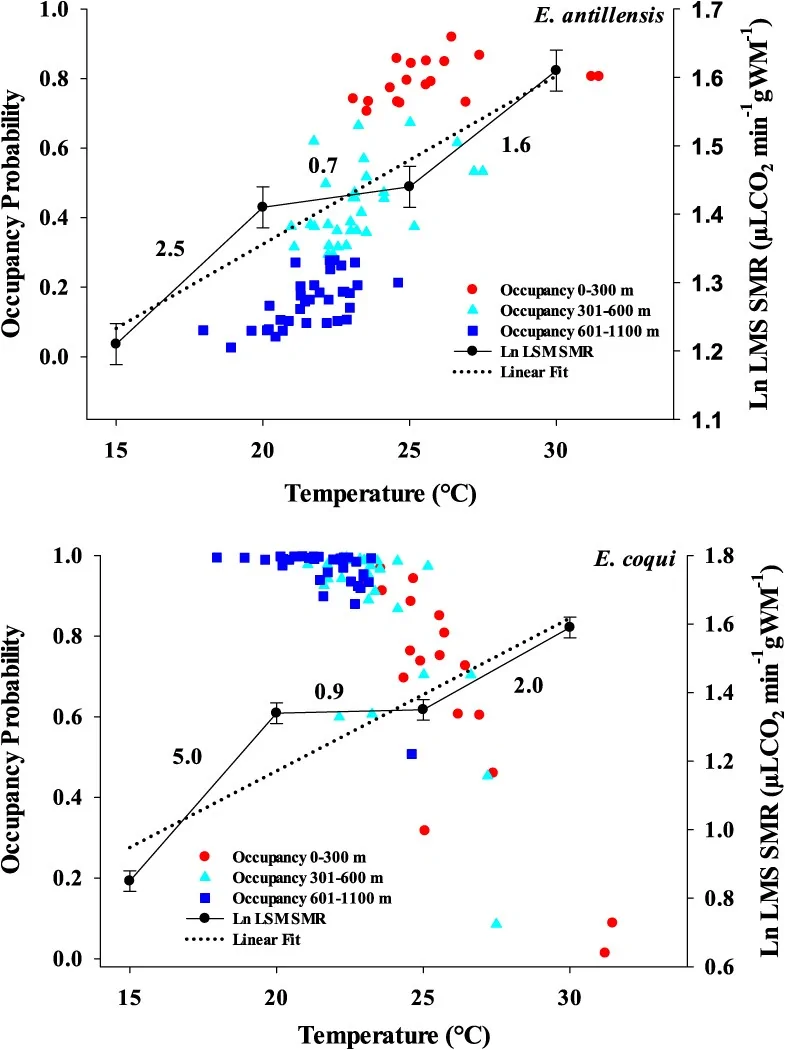
Routine metabolism of *E. antillensis* and *E. coqui* measured in this study (redrawn from Fig. 4), integrated with occupancy probabilities based on temperature (°C) and elevation (m) generated from data collected by Rivera-Burgos *et al.* (2021). Circles depict occupancy levels from sea level to 300 m (0–300), triangles from 301 to 600 m, and squares from 601 to 1100 m.

## Discussion

### Overview

A species’ risk of extinction under future climate conditions will be shaped, in part, by their physiological traits (Greenberg and Palen, 2021). Therefore, there is a growing interest in evaluating the metabolic and physiological responses of amphibians to rising temperature regimes as these responses are central to their energy budget, scale up to a species’ abundance and distribution, and are essential to formulate appropriate adaptation strategies (McDonald-Madden *et al.*, 2011; Morelli *et al.*, 2016; Burrowes *et al.*, 2021).

### Role of diel cycle and temperature on routine metabolic rates

Metabolic rates for both *E. antillensis* and *E. coqui* reflected diel cyclicity, peaking during nighttime activity at 20 and 25°C. Lower diurnal routine metabolism may be the result of decreased locomotor activity, as well as biochemical control of metabolic pathways during inactive periods (Adamovich *et al.*, 2019). For these species, reducing energy expenditures in diurnal retreats could result in a positive energy balance. Reduced metabolic demands while in diurnal retreats is a documented behaviour in small ectotherms due to their high surface area-to-volume ratio (Gillooly *et al.*, 2001). Consequently, reducing maintenance costs during the day allows for a positive energy balance to fuel active foraging and territorial behaviours during nocturnal periods. Such diel metabolic adaptations allow assimilated energy to fuel biosynthetic pathways during inactive periods. However, as thermal regimes are forecasted to rise in Puerto Rico (Bowden *et al.*, 2021), the metabolic costs of maintaining physiological function are likely to increase, unless thermal refugia or behavioural thermoregulation allow compensation, to an extent. This could diminish the energy assimilated and stored during inactive periods, constraining the energy balance and challenging these species as habitat temperatures approach or exceed their physiological limits (Hillman *et al.*, 2009; Huey *et al.*, 2012).

### Thermal performance curves of routine metabolic rates

Experimental trials showed an overall increased metabolic rate with increasing temperature for *E. antillensis*, *E. coqui*, and *E. juanariveroi*. Both *E. antillensis* and *E. coqui* displayed a linear relationship between metabolic rate and temperature, with no apparent reduction in metabolic performance in the highest temperature treatments. In contrast, routine metabolism of *E. juanariveroi* also increased linearly with temperature, but showed decreased performance beyond 25°C. Indeed, the ABT in routine metabolism was found to be at 25°C for this species, suggesting a shift in the processes controlling metabolic pathways around these temperatures. Although instructive, due caution must be exercised when interpreting inflection points such as the ABT (Schulte, 2015). We acknowledge that four treatment temperatures (15, 20, 25, and 30°C) is a low number of points for breakpoint analyses. For this reason, we also calculated Q_10_ values, and values for *E. juanariveroi* also suggested that there is a decrease in metabolism at 25–30°C (Table 1). This kind of assessment was not possible for *E. wightmanae* as RMR values at 25°C were unreliable and no individuals survived at 30°C. We recommend that future work for all *Eleutherodactylus* species use narrower temperature increments to permit more robust and precise estimation of thermal transitions. Additionally, assessments should include inter-population comparisons to be more instructive and improve the ability to generalize findings for species throughout their range.

### Metabolic adaptations to temperature in low-elevation species

*E. antillensis* and *E. juanariveroi* have strong affinities for low-elevation habitats characterized by warmer temperatures (Ríos-López *et al.*, 2014; Senior *et al.*, 2018; Rivera-Burgos *et al.*, 2021). Yet, their TPC responses differed in marked ways (Fig. 5). The TPC RMR for *E. antillensis* affirmed its ability to occupy warmer, lowland areas (Campos-Cerqueira *et al.*, 2021; Rivera-Burgos *et al.*, 2021). In contrast, *E. juanariveroi* responded linearly up to 25°C, close to the *in situ* temperature recorded where specimens were collected (24.27°C ± 0.09; Diez, 2025), but decreased at 30°C. It is noteworthy that Rohr *et al.* (2010), Narayan *et al.* (2012), Navas *et al.* (2013) and Schulte (2015) suggested that warm-tolerant species may not be able to efficiently harness the assimilated energy into useful cellular energy when faced with increased temperatures. This situation compromises the energy available for whole-organism processes such as growth and reproduction (Somero *et al.*, 1996; Wintrode and Arnold, 2001). Thus, warm-tolerant or warm-adapted species might have to contend with increased cost of maintenance due to the temperature-dependent nature of the biochemical reactions as appears to be the case with *E. juanariveroi* (Cossins and Bowler, 1987; Somero *et al.*, 1996; Morey and Reznick, 2004; Tattersall *et al.*, 2012; Schulte, 2015).

It is also important to keep in mind that increases in temperatures could exacerbate the costs inferred by our TPC RMR results in other ways. For example, species like *E. antillensis* may face challenges maintaining proper hydration levels (Pough *et al.*, 1984). When compared to *E. coqui*, *E. antillensis* could rehydrate much faster than either lowland or highland *E. coqui* (Berkum *et al.*, 1982). Efficient rehydration is crucial to sustain physiological activities such as locomotion (Greenberg and Palen, 2021), and to reduce skin desiccation. Additionally, energetically demanding immune responses to pathogens such as chytridiomycosis, a fungal disease caused by a chytrid fungus (*Bd, Batrachochytrium dendrobatidis*), also pose further challenges to the energy budget of warm-adapted amphibians (Longo *et al.*, 2009; Joglar *et al.*, 2011).

### Thermal sensitivity and survival of high-elevation specialists

As hypothesized, RMR experiments confirmed that *E. wightmanae*, a high-elevation specialist, exhibited routine metabolism similar to its widely distributed counterparts in the 15–20°C range. However, warmer temperatures led to unreliable data points at 25°C. The observation that none of the specimens survived at 30°C matches similar findings reported for another high-elevation specialist, *E. portoricensis* (Beuchat *et al.*, 1984; Delgado-Suazo and Burrowes, 2022). We note that these responses align with patterns observed in other high-elevation, cold-adapted species, including salamanders, which are highly susceptible to thermal stress and metabolic disruption (Homyack *et al.*, 2010; Gangloff and Telemeco, 2018; Markle and Kozak, 2018; Riddell *et al.*, 2018). Across taxa, montane organisms adapted to cold, stable environments exhibit similar vulnerabilities, highlighting a consistent trend of heightened sensitivity to climate change-induced warming. Highlighting these parallels broadens the scope of this study and underscores the broader vulnerability of montane specialists to climate change. Additionally, the influence of diel cycles on energy expenditure during inactive periods further exacerbates this vulnerability, as higher metabolic demands during warmer daytime conditions may reduce energy available for survival and reproduction.

The specific biochemical responses and molecular underpinnings occurring in *E. wightmanae* and other *Eleutherodactylus* high-elevation species will likely be affected by continued increases in temperatures at high elevations in Puerto Rico (Bowden *et al.*, 2021; Runkle *et al.*, 2022) and await future research to be elucidated. Further work with *E. wightmanae* should focus on increasing the resolution of the metabolic rate curve developed in this study, with particular emphasis on mechanistic assessment of energy production (i.e. mitochondrial thermal performance; Salin *et al.*, 2012) and on evaluating how diel cycles influence metabolic demands during both active and inactive periods.

### Conservation implications

Physiological studies such as the one presented herein are useful to help elucidate conditions that warrant consideration of adaptation strategies such as managed translocations and identification of climate-resilient habitats (McDonald-Madden *et al.*, 2011; Morelli *et al.*, 2016; Barrows *et al.*, 2020). Our work underscores the importance of climate-resilient habitats, particularly those retaining cooler microhabitats, to buffer against temperature extremes that support high-elevation specialists like *E. wightmanae*. Another valuable endeavour is to integrate information on RMRs, including diel metabolic cycles, with field studies to gain insights into how physiological processes manifest themselves at higher levels of ecological phenomena, such as abundance and distribution (Connolly *et al.*, 2017). An example of this application is illustrated by *E. antillensis* and *E. coqui*. These species have contrasting distribution patterns: *E. antillensis* had a higher occurrence at low elevations, whereas *E. coqui* has an affinity for high-elevation habitats (Fig. 6; Rivera-Burgos *et al.*, 2021). Interestingly, the low sensitivity of RMR at temperatures linked to moderate to high occupancy suggests potential plasticity in routine metabolic demands with increasing temperature. A plausible interpretation of these relationships is that both species’ thermotolerant physiology reflects their ability to withstand a wider range of temperatures, which is a contributing factor for their widespread distribution on the island (Campos-Cerqueira *et al.*, 2021). Although instructive, this integration will benefit from future studies disentangling the role of elevation and temperature in RMR, and the sublethal effects of increased metabolism for these and other eleutherodactylids. Interestingly, and plausibly due to its thermotolerant physiology, *E. coqui* is the only eleutherodactylid that has been reported in multiple invasions in Central America, Florida, and Hawaii (Austin and Schwartz, 1975; Wilson and Porras, 1983; Stewart and Woolbright, 1996; Pitt *et al.*, 2012; Westrick *et al.*, 2022). Insights from integrating information, as illustrated above, could be valuable to strengthen other fields in ecology such as range dynamics (MacKenzie *et al.*, 2006). Modellers could benefit from insights into the relationships among physiological processes, projected spatial and temporal environmental changes, and distribution to advance stronger predictions about range expansion or contraction (Werner *et al.*, 2009; Walls and Gabor, 2019; Connolly and Popesku, 2024).

## Data Availability

Data and metadata are available through the ScienceBase repository at the following links: https://www.sciencebase.gov/catalog/item/65009778d34ed30c2057f6e5  https://www.sciencebase.gov/catalog/item/5fd26673d34e30b91239be52, and https://www.sciencebase.gov/catalog/item/63372b6cd34e900e86cb48f4.
